# Economic Impact of Implementing Malnutrition Screening and Nutritional Management in Older Adults in General Practice

**DOI:** 10.1007/s12603-020-1331-6

**Published:** 2020-02-07

**Authors:** Fiona Brown, G. Fry, A. Cawood, R. Stratton

**Affiliations:** 1 Department of Nutrition and Dietetics, Gloucestershire Hospitals NHS Foundation Trust, Cheltenham, Gloucester, GL51 7AN UK; 2 Faculty of Medicine, Institute of Human Nutrition, Mail point 113, Southampton General Hospital, Tremona Road, Southampton, SO16 6YD UK; 3 Medical Affairs, Nutricia Ltd, White Horse Business Park, Trowbridge, Wiltshire, BA14 0XQ UK

**Keywords:** Malnutrition, general practice, oral nutritional supplements, dietary advice, pathway

## Abstract

**Objectives:**

Malnutrition is a common and significant public health problem, especially for older adults, as the consequences are costly. National guidelines (NICE CG32/QS24) highlight the need to identify and manage malnutrition, the implementation of which was deemed “high impact to produce cost savings”. The ‘Malnutrition Pathway’, endorsed by NICE and other professional bodies, is a practical evidence-based guide to help community healthcare professionals (HCP) to implement guidance on malnutrition management. Published evaluations of its use are needed.

**Design:**

This service evaluation in older adults assessed the impact of implementing the ‘Malnutrition Pathway’ on health care use and costs, as well as the acceptability of the management strategies and effect on malnutrition risk.

**Setting:**

5 GP surgeries in Gloucestershire.

**Participants:**

163 older adults (80±9 years) with a range of primary diagnoses, living in their own home, were screened using the Malnutrition Universal Screening Tool (‘MUST’) (n50 low risk (LR); n41 medium risk (MR); n72 high risk (HR)). All patients were managed according to risk (LR: no further management; MR: dietary advice (DA); and HR: DA plus two oral nutritional supplements (ONS) (1 serve 300kcal, 18g protein; 125ml).

**Measurements:**

At each review (6weeks, 3 and 6 months), ‘MUST’ score, compliance and satisfaction to their management plan were recorded. Healthcare use was collected from GP records 6 months before and after implementation of the pathway. A simple cost analysis was completed.

**Results:**

Implementing appropriate management of malnutrition led to significant reductions in hospital admissions (p=0.028), length of hospital stay (p=0.05), GP visits (p=0.007) and antibiotic prescriptions (p=0.05). Over 6 months, the costs to manage malnutrition (HCP time, ONS) were more than offset by the savings associated with these reductions in health care use (per patient savings of −£395.64 MR+HR; −£997.02 HR). The proportion of individuals at risk of malnutrition reduced over time, and patients reported being satisfied with the DA (97%) and ONS (96%), consuming 90% of their ONS prescription.

**Conclusion:**

Managing malnutrition significantly reduces healthcare use, with a positive budget impact, in older malnourished patients in primary care. This represents an opportunity to improve patient care with benefit on health care spend.

## Introduction

Malnutrition is a common and costly problem, with health and social care costs estimated to be around £23.5 billion per year in the UK (1), around 15% of the health and social care budget (2). Consequences of malnutrition include increased complications, GP visits, and hospital readmissions, important in the community where most individuals reside, with prevalence in General Practice reported between 7 and 10% (3, 4). It is important to manage malnutrition in the right way as the largest costs are associated with the extensive consequences of undetected and unmanaged malnutrition. Indeed the annual health and social care costs are estimated to be nearly 4 times greater for a malnourished patient (£7408), than a non-malnourished patient (£2155) (1).

Malnutrition should be identified with a screening tool like the ‘Malnutrition Universal Screening Tool’ (‘MUST’) http://www.bapen.org.uk/screening-and-must/must-calculator, which requires measures of height, weight and BMI, and managed according to national guidance (5, 6). Improving management can result in significant cost savings of over £71,800 per 100,000 population (5). To support community healthcare professionals a practical guide “Managing Adult Malnutrition in the Community” (“Malnutrition Pathway”) was developed which includes a pathway for management and guidance on the appropriate use of oral nutritional supplements (ONS) (7). It was produced by a multi-professional consensus panel and endorsed by 10 professional bodies including the Royal College of General Practitioners, Royal Pharmaceutical Society, Royal College of Nursing, Primary Care Pharmacists Association, British Dietetic Association as well as NICE (https://www.malnutritionpathway.co.uk/library/managing_malnutrition.pdf).

Although there is evidence to support the use of nutrition support strategies including DA and ONS (8–10) there is limited evidence of the impact of implementing both identification and appropriate management of malnutrition in practice using this malnutrition pathway.

The primary aim of this local service evaluation was to assess the economic impact of implementing the Malnutrition Pathway in older adults (≥65 years) in General Practice, as well as assessing its acceptability and effect on overall malnutrition risk.

## Methods

**Figure 1:**
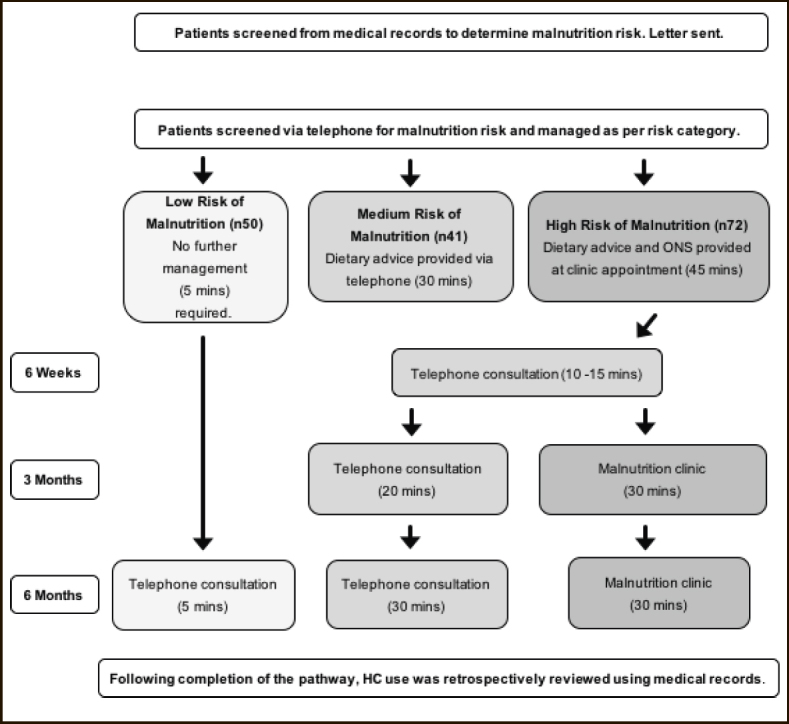
Flow diagram describing service evaluation methods

163 older adults (≥65years), from 5 GP surgeries in Gloucestershire took part in this dietetic led service evaluation and were managed for malnutrition risk according to the Malnutrition Pathway (Figure 1). Malnutrition risk was identified by the dietitian using the Malnutrition Universal Screening Tool (‘MUST’). This screening tool is an integral part of the Malnutrition Pathway and is primarily comprised of: step 1 BMI, step 2 unintentional weight loss in the last 3–6months and step 3 if there is no, or likely to be no nutritional intake for more than 5 days as the patient is acutely ill (which is unlikely to occur outside of hospital). ‘MUST’ scores can range from 0–6, with 0 being low risk, 1 medium risk and 2+ high risk. Individuals with a ‘MUST’ score of 1 or more (medium or high risk of malnutrition) are classified as “at risk” of malnutrition and require oral nutrition support (A grade recommendation NICE CG32), these individuals are the “pathway group” in this service evaluation. Management strategies were implemented by the dietitian. Low risk patients (LR, n=50) (‘MUST’ Score 0) received routine care (no intervention for malnutrition) and were rescreened 6 months later over the telephone. Medium risk patients (MR, n=41) (‘MUST’ Score 1), were provided with dietary advice over the telephone. Dietary advice was provided by the dietitian based on Gloucestershire Hospitals NHS Foundation Trust’s ‘Food First’ diet sheets and patients were reviewed by the dietitian by telephone after 6 weeks. High risk patients (HR, n=72) (‘MUST’ Score 2+) were invited to attend a clinic appointment with the dietitian at their surgery where they were provided with DA and a prescription for two ready-made liquid ONS (Fortisip Compact Protein, Nutricia; 2 bottles, 600kcal, 36g protein, range of vitamins and minerals).

For all patients at risk of malnutrition (pathway group) data was collected at the initial appointment, at 6 weeks (±2 days), 3 months (±2 days), and 6 months (±2 days). Age, gender, primary diagnosis and height were recorded at the initial appointment only. At each review ‘MUST’ score was documented, along with details of compliance and satisfaction with their dietary management and ONS. All pathway group patients were asked the same series of questions regarding the dietary advice they received; were they following it, have they made changes to their diet (both yes/no questions), how easy was it to follow and how satisfied they were with the DA (answered using a 5-point likert scale). The HR patients were asked further questions regarding ONS; were they taking their ONS, how easy was it to take, and how satisfied they were with the ONS. For all telephone appointments measures were self-reported, for those attending clinic weight and height were measured to the nearest 0.1kg (Marsden (MS-4202L) calibrated scales) and 1cm (Leicester Height Measure) respectively. If at any of the review points, a patient was no longer at risk of malnutrition they were discharged back into usual care and were not reviewed further.

Following the 6 month review a retrospective audit of the patient’s healthcare use (number of hospital admissions, length of hospital stay (days), number of GP appointments, total number health care professional visits (including GP, nurse, allied health and hospital doctor appointments) and number and type of antibiotic prescriptions) was collected by the dietitian onto standardised forms from the information available in the electronic patient record in each GP surgery. Healthcare use was captured for the 6-month period prior to enrolment onto the Malnutrition Pathway and for the 6-months following its implementation.

A simple cost analysis was undertaken on a per patient basis comparing cost of healthcare resource used 6 months before and after implementation of the Malnutrition Pathway. Individual costs (Table 1) included costs for dietetic time, GP consultation, HCP contacts, antibiotic prescriptions, and hospital admissions which were obtained from Unit Costs of Health and Social Care for 2016 and Department of Health, Reference Costs 2015–2016 (11, 12). The cost for ONS was based on 2 bottles of Fortisip Compact Protein per day for 14 weeks (average length of prescription during the service evaluation) (13). The cost analysis was completed in two ways using, [1] the cost per day of hospital stay and [2] cost of an average hospital admission.

**Table 1:** Table summarising costs used in the simple cost analysis

**Resource**	**Cost per patient**
Dietitian	£63 per MR patient and £84 per HR patient (average taken on 90 mins being spent with MR patient (4 telephone calls) and 120 mins being spent with a HR patient (3 clinic appointments and 1 telephone call) (Band 6 £42 per hour) [section II, 9, page 137] (12)
GP consultation for ONS prescription	£36 per HR patient [section II, 10.3, page 145] (12)
ONS prescription	£4.00 per day for 14 weeks (based on 2 x low volume, high protein ONS per day (Fortisip Compact Protein, Nutricia) for average length of prescription) (13)
Hospital admission	£2679 (average cost taken from the cost of an elective admission £3749 and non-elective admission £1609) [chapter 2, page 10] (11)
Cost of hospital stay	£306 per day [chapter 2, page 10] (11)
HCP contact	£40 (estimated cost assigned based on average of wide range of HCPs) [section IV, 13, page 194] (12)
Antibiotic prescription	£28 unit cost of prescription [section II, 10.3, page 145] (12)

MR-medium risk, HR-high risk, HCP-healthcare professional, ONS-oral nutritional supplement

### Data analysis and Statistics

Data was collated and analysed using IBM SPSS Statistics package version 23 (IBM Corporation, New York, US). Data was presented as mean values ± standard deviation (SD) unless stated otherwise. A one-way ANOVA was used to compare healthcare use (number of healthcare visits, number of hospital admissions, number of antibiotic prescriptions and total length of hospital stay (days)) from 6 months before and 6 months after implementation of the pathway. Paired t-tests were used to make comparisons of the same outcomes at 2 time points, within the different malnutrition risk groups. As this is a small service evaluation power calculations were not undertaken to determine sample size. A pragmatic approach was taken to recruit as many suitable individuals within the dietetic resource available.

## Results

163 patients (Table 2) (58% female, mean age 80±9 years, weight 57.2±14.3kg, BMI 21.0±4.3kg/m^2^) had a wide range of primary diagnoses including respiratory (24%; majority COPD (85%)); cardiovascular (CVS) (16%); musculoskeletal (14%); endocrine (11%); genitourinary (GU) (10%), gastrointestinal (GI) (7%), neurological (non CVA) (5%), and haematology (2%). Of the total group (n163), 113 (MR+HR) followed the Malnutrition Pathway (pathway group). All 72 patients in the HR group were prescribed ready-made liquid ONS for an average of 14.2±8.6 weeks and received DA. All 41 patients in the MR group were provided with DA and no nutrition support actions were taken with the patients in the LR group (n50).

**Table 2:** Initial characteristics of patients before implementation of the ‘Malnutrition Pathway’, whole group and individual malnutrition risk groups, mean ± s.d

	**Whole group n=163**	**Pathway group MR+HR n=113**	**HR n=72**	**MR n=41**	**LR n=50**	**p value**
Age, years	80±9	83±8	83±7	82±9	75±6	0.000^a^
Weight, kg	57.2±14.3	51.1±10.1	48.6±9.1	55.5±10.2	71.0±12.9	0.000^a^
BMI, kg/m^2^	21.0±4.3	19.1±2.9	18.3±3.1	20.5±2.0	25.4±3.8	0.000^a^
Gender:						0.809^b^
- male	68 (42%)	46 (41%)	28 (39%)	18 (44%)	22 (44%)	
- female	95 (58%)	67 (59%)	44 (61%)	23 (56%)	28 (56%)	
Primary Medical Diagnosis						NS
- Respiratory	39 (24%)	37 (33%)	26 (36%)	11 (27%)	2 (4%)	
- Cardiovascular	26 (16%)	16 (14%)	8 (11%)	8 (20%)	10 (20%)	
- Musculoskeletal	23 (14%)	15 (13%)	12 (17%)	3 (7%)	8 (16%)	
- Endocrine	18 (11%)	9 (8%)	4 (6%)	5 (12%)	9 (18%)	
- Genitourinary	16 (10%)	13 (12%)	8 (11%)	5 (12%)	3 (6%)	
- Gastrointestinal	12 (7%)	5 (4%)	4 (6%)	1 (2%)	7 (14%)	
- Neurological (non-CVA)	8 (5%)	8 (7%)	5 (7%)	3 (7%)	0 (0%)	
- Mental Health	4 (3%)	3 (3%)	3 (4%)	0 (0%)	1 (2%)	
- Haematology	3 (2%)	3 (3%)	1 (1%)	2 (5%)	0 (0%)	
- No Diagnosis	14 (9%)	4 (4%)	1 (1%)	3 (7%)	10 (20%)	

^a^ 1-way ANOVA, comparison of risk groups, ^b^ Chi Square Test (non-parametric data); LR-low risk, MR-medium risk, HR-high risk.

### Health care use

When comparing 6 months before with 6 months after implementation of the Malnutrition Pathway overall there were significant differences in healthcare use in the pathway group (MR+HR), and those at HR only, but not in the MR patients (Table 3). In those at risk of malnutrition (MR+HR) there was a 49% reduction in hospital admissions (p=0.028), 48% reduction in length of hospital stay (p=0.05), 21% fewer GP appointments (p=0.007), 30% fewer antibiotic prescriptions (p=0.05), and 13% less healthcare professional contacts (p=0.103). In the HR group, there was a 62% reduction in hospital admissions (p=0.005), 67% reduction in length of hospital stay (p=0.004), 25% reduction in number of GP appointments (p=0.006); 39% reduction in number of antibiotic prescriptions (p=0.04) and 21% reduction in total number of healthcare professional visits (p=0.04) (Table 3). Six months after implementation of the pathway the proportion of patients admitted to hospital was significantly lower (26.5% to 12.4%; p=0.034), as was the proportion prescribed antibiotics (45.1% to 27.4%; p=0.024), there was also a 15% reduction in the proportion of patients visiting their GP although not significant (p=0.739) (data not shown). A review of the LR group of patients at 6 months found 6 patients were at risk of malnutrition, these patients were referred on for appropriate follow up, there was no significant changes in healthcare use in the LR group.

**Table 3:** Health care use in the 6 months before and after implementation of the ‘Malnutrition Pathway’

	**6 months before**		**6 months after**			
	**Mean**	**S.D.**	**Mean**	**S.D.**	p value*	**% change**
Number of Hospital Admissions:						
MR+HR	0.43	0.85	0.22	0.62	0.028	−49%
MR	0.16	0.50	0.21	0.62	0.661	+39%
HR	0.60	0.98	0.23	0.62	0.005	−62%
Length of Hospital Stay:						
MR+HR	4.59	10.92	2.37	6.92	0.050	−48%
MR	1.16	4.90	2.61	7.94	0.327	+125%
HR	6.77	12.98	2.22	6.24	0.004	−67%
Number of GP contacts:						
MR+HR	6.44	5.50	5.12	4.14	0.007	−21%
MR	5.08	5.04	4.55	4.07	0.468	−10%
HR	7.3	5.65	5.48	4.17	0.006	−25%
Total number of HCP^ visits:						
MR+HR	11.38	9.92	9.92	1.00	0.103	−13%
MR	8.74	6.56	9.16	12.03	0.818	+5%
HR	13.05	11.29	10.37	9.70	0.040	−21%
Number of Antibiotic Prescriptions:						
MR+HR	0.84	1.21	0.59	1.01	0.050	−30%
MR	0.79	1.36	0.68	1.25	0.593	−14%
HR	0.87	1.11	0.53	0.83	0.040	−39%

MR-medium risk, HR-high risk, HCP-healthcare professional; *Paired †-test4; ^Total number of HCP visits includes GP visits, nurse visits, hospital doctor and allied health professional visits; No significant changes seen in the LR (low risk) group (data not shown)

### Simple cost analysis

The simple cost analysis, using standard costs (Table 1) was undertaken on a per patient basis 6 months pre- and postimplementation of the Malnutrition Pathway. For the pathway group overall costs were based on the reductions seen in: (a) length of hospital stay (2.22 days @ £306 per day = −£679.32, (b) total number of HCP contacts (1.46 contacts @ £40 = −£58.40) and (c) number of antibiotic prescriptions (0.25 less @ £28 = −£7.00), as well as the increased costs per patient to implement the pathway, including ONS prescription in the HR group (+£249.76), dietetic time in MR+HR group (+£76.38); and GP prescribing time in the HR group (+£22.94). Overall the cost to implement the pathway per patient over 6 months was +£349.08 and the savings from reduction in health care use were −£744.72, with an overall cost saving per patient for 6 months of −£395.64. When using an average hospital admission cost (Table 1) rather than length of stay cost, a similar overall cost saving was seen (−£278.91). For the HR group only, based on the reduction in health care use seen, the savings included (a) length of hospital stay (4.55 days @ £306 = −£1392.30, (b) total number of HCP contacts (2.68 contacts @ £40 = −£107.20) and (c) number of antibiotic prescriptions (0.34 less @ £28 = −£9.52). The increased costs to manage those at high risk only included: ONS prescription (+£392); dietetic time (+£84); and GP prescribing time (+£36). Overall the savings per patient over 6 months in the high risk only group were £-997.02. As before if the model was based on average admission cost rather than length of stay the overall savings are −£595.95.

### Malnutrition risk

Over the period of the evaluation there was an overall improvement in malnutrition risk. Using available risk data at 6 months, of those that were all initially MR, 66.6% had been previously discharged from dietetic care at an earlier visit due to being LR, 13.3% were now LR, 16.6% remained MR, 2.4% had become HR. Similarly, of those that were initially HR, 27.5% had been previously discharged from dietetic care at an earlier visit due to being LR, 7.8% were now LR, 17.6% were now MR, and 47% remained HR.

### Acceptability of oral nutrition support strategies and compliance to ONS

The majority (81%) of all patients on the pathway found the dietary advice they were given easy or very easy to follow and 97% of patients were satisfied or very satisfied with the dietary advice. 92% of the patients also given ONS (the HR group) found that the ONS were easy or very easy to take and 96% were satisfied or very satisfied with the ONS. Patients reported taking a mean of 90% of the ONS they had been prescribed (Table 4).

**Table 4:** Satisfaction with oral nutrition support over the 6 month evaluation period

**Questions for pathway group (n113)**	**Mean %**
*Following Dietary Advice?*	
Yes every day	66.0%
Sometimes	13.4%
No not at all	20.7%
*Made Changes to Diet?*	
Yes	47.6%
No	52.4%
*Ease to Follow Diet Advice?*	
Very Easy	20.4%
Easy	60.4%
Neither Easy or Difficult	8.5%
Difficult	10.4%
Very Difficult	0.3%
*Overall satisfaction with Dietary Advice?*	
Very Satisfied	51.1%
Satisfied	46.1%
Neither satisfied or dissatisfied	2.8%
Slightly dissatisfied	0.0%
Very dissatisfied	0.0%
Questions for HR only* (n72)	**Mean %**
*Taking ONS?*	
Yes every day	85.4%
Sometimes	12%
No not at all	2.6%
*Ease to Take ONS?*	
Very Easy	60.7%
Easy	31.4%
Neither Easy or Difficult	2.2%
Difficult	5.7%
Very difficult	0.0%
*Overall satisfaction with ONS?*	
Very Satisfied	76.7%
Satisfied	18.8%
Neither satisfied or dissatisfied	4.1%
Slightly Dissatisfied	0.5%
Very dissatisfied	0.0%

HR High Risk; *All participants were asked about DA and then those at High Risk (HR) the additional questions about ONS

## Discussion

This service evaluation demonstrates practically the positive impact of following the Malnutrition Pathway for patients at risk of malnutrition in primary care, over a short period of time. It highlights the importance of screening and appropriate management which includes using oral nutrition support and reviewing patients against goals. It highlights the greatest impact for both the patient and healthcare economy is in the management of those at HR, who received DA and high protein ready to drink ONS. Overall patients at HR achieved the greatest reductions in hospital admissions, length of stay, GP contacts, and antibiotic prescriptions, all of which were significantly reduced. Alongside the economic benefits, malnutrition risk was reduced and patients reported being highly satisfied with the interventions (DA and ONS), and had excellent compliance to ONS.

Overall the costs associated with managing malnutrition (cost of screening, HCP time and ONS), were more than offset by the savings associated with these reductions in health care use with overall cost savings (per patient over 6 months) estimated between −£278.91 and −£395.64 in the pathway group (MR+HR), and between −£595.95 and −£997.02 for those at HR. When considering a simple budget impact model, extrapolating these savings to a representative population of 100,000 people who are older, live in the community and are at risk of malnutrition, could mean potential cost savings of between +£344,733 and + £641,084 (Table 5).

**Table 5:** Theoretical calculation of budget impact per 100,000 population

**Population**	**100000**
Percentage who are ≥65years UK (18.2%) (26)	18200
Percentage living in the community (93%) (27)	16926
Percentage at risk of malnutrition (7.3%) (4)	1236
Cost saving based on local implementation savings for those at risk of malnutrition (MR+HR; −£278.91^1^ to −£395.64^2^)	£344,733 to £489,011
Percentage at high risk of malnutrition (3.8%) (4)	643
Cost saving based on local implementation savings for those at high risk of malnutrition (HR; −£595.95^1^ and −£997.02^2^)	£383,196 to £641,084

MR-medium risk, HR- high risk; ^1^ Overall cost saving over 6 months, costs to implement - savings in health care use; using length of hospital stay; ^2^ Overall cost saving over 6 months, costs to implement - savings in health care use; using average hospital admission cost

The reason for the greatest benefit in the HR group may be linked to them receiving a combined approach to nutrition support. These patients likely had a poor appetite and dietary intake due to their disease, making improving intake of energy, protein and micronutrients key. Indeed the majority of patients received DA and ONS high in protein, based on clinical benefits and the increased protein needs of the patients (10, 14) however this may mean the benefits seen in this service evaluation might not be replicated with other types of nutrition support.

Malnutrition screening and management in primary care is a significant clinical issue as recently highlighted (15–17). It is well known that oral nutritional support, including DA and ONS, has nutritional benefits and has been shown to improve intake and weight with benefits being the greatest when the interventions are used in combination (8). More specifically, ready to drink ONS have consistently been shown to improve energy and weight, improving recovery and reducing health care use (9, 10, 18, 19). These outcomes are also consistent with other real-world evidence from care homes (20) and the results from a recent large randomised controlled trial conducted in primary care (21–23).

To our knowledge this is the first service evaluation to assess in practice the effect of managing disease related malnutrition in older people registered with their GP. It not only provides local data of the potential benefit but also provides evidence to demonstrate that the theoretical high impact cost savings calculated by NICE are achievable in the real world.

It should be considered that there are also limitations; it is based on a relatively small number of individuals residing in one local area and has not been undertaken in a randomised manner or with a control group as this is an evaluation of service, real world evidence. The evaluation was undertaken by a registered dietitian, while not a limitation, there are currently insufficient dietitians to support all malnourished community patients, so it maybe that the management of malnutrition in the community be undertaken by other healthcare professionals following the Malnutrition Pathway under the guidance of dietitians. Despite these points the results seen are similar those obtained previously from other service evaluations and randomised trials that produce cost savings (20, 24, 25).

The consequences of untreated malnutrition are costly, implementing the Malnutrition Pathway into primary care including identification and appropriate management is quite simple, has high impact for savings in the short term and follows NICE guidance. With ever-growing cost pressures, an approach to proactively identify and manage malnutrition may be an ideal area to focus especially as it links with so many NHS priorities.
